# The disease burden and its distribution characteristics of clonorchiasis in Guangdong Province, Southern China

**DOI:** 10.1186/s13071-024-06425-z

**Published:** 2024-08-21

**Authors:** Datao Lin, Zhuohui Deng, Zebin Chen, Kefeng Jiang, Qiming Zhang, Wenjing Zhou, Qixian Zhang, Jun Liu, Zhongdao Wu, Lan Guo, Xi Sun

**Affiliations:** 1 Department of Parasitology, Key Laboratory of Tropical Disease Control (Ministry of Education), Zhongshan School of Medicine, Sun Yat-Sen University, Guangzhou, 510080 China; 2 Guangdong Provincial Center for Disease Control and Prevention, WHO Collaborating Centre for Surveillance, Research and Training of Emerging Infectious Diseases, Guangzhou, 511430 China; 3 Center of Hepato-Pancreato-Biliary Surgery, The First Affiliated Hospital, Sun Yat-Sen University, Guangzhou, 510080 China; 4 Department of Medical Statistics and Epidemiology, School of Public Health, Sun Yat-Sen University, Guangzhou, 510080 China; 5 Department of Gastroenterology, The Third Affiliated Hospital, Sun Yat-Sen University, Guangzhou, 510630 China

**Keywords:** Clonorchiasis, Disease burden, Prevalence, DALY, Cost of illness

## Abstract

**Background:**

Clonorchiasis has significant socioeconomic importance in endemic areas; however, studies investigating the disease burden in specific sub-regions are lacking. This study aims to address the gap by quantifying the current disease burden caused by clonorchiasis in Guangdong province and assessing its distribution characteristics.

**Methods:**

Comprehensive measures, including prevalence rates, disability-adjusted life years (DALYs), and direct medical costs, were used to assess the disease burden of clonorchiasis. To estimate the prevalence rate, the number of infections was divided by the examined population, based on the annual surveillance data on clonorchiasis cases during 2016–2021. The calculation of DALYs was based on the epidemiological parameters according to the definition issued by the World Health Organization. Cost data of clonorchiasis were utilized to quantify the direct medical costs. The distribution characteristics of disease burden were assessed through comparisons of groups of population defined by geographic area, time, and characteristics of people.

**Results:**

In 2021, clonorchiasis posed a significant disease burden in Guangdong Province. The prevalence rate was found to be 4.25% [95% CI (4.02%, 4.49%)], with an associated burden of DALYs of 406,802.29 [95% CI (329,275.33, 49,215,163.78)] person-years. The per-case direct medical costs of patients with clonorchiasis were estimated to be CNY 7907.2 (SD = 5154.4). Notably, while the prevalence rate and DALYs showed a steady decrease from 2016 to 2020, there was a rising trend in 2021. Spatial clustering of clonorchiasis cases and DALYs was also observed, particularly along the Pearl River and Han River. This suggests a concentration of the disease in these regions. Furthermore, significant differences in prevalence rates were found among various demographic groups, including sex, age, occupation, and education level. Additionally, patients with longer hospital stays were more likely to incur higher direct medical costs.

**Conclusions:**

The burden of clonorchiasis in Guangdong Province remains high, despite significant progress achieved through the implementation of the prevention and control programs. It is suggested that measures should be taken based on the distribution characteristics to maximize the effectiveness of prevention and control, with a primary focus on key populations and areas.

**Graphical Abstract:**

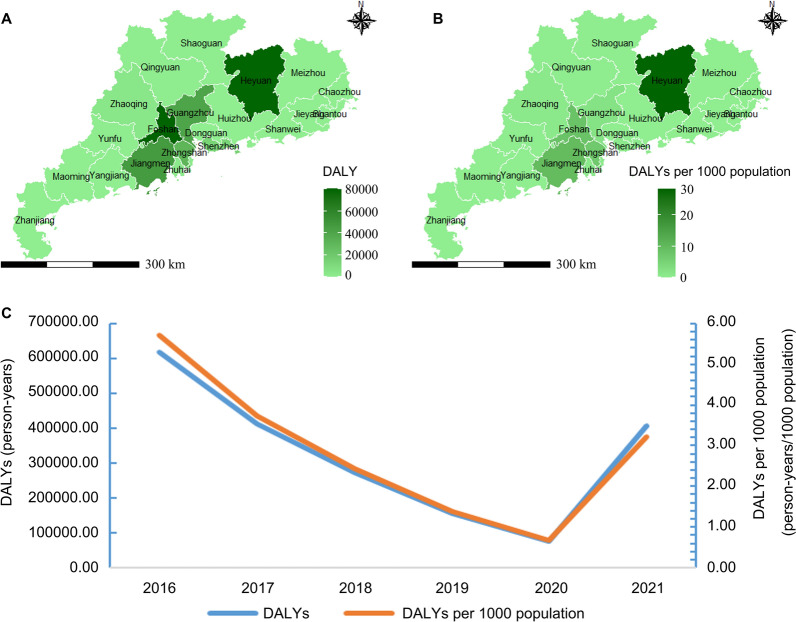

## Background

Clonorchiasis, caused by *Clonorchis sinensis*, is a significant neglected tropical disease with features of low mortality and high disability [1, 2]. This liver fluke infection is predominantly prevalent in areas with habits of eating raw fish and carries significant socioeconomic implications in endemic areas [3, 4]. It has been estimated that the total economic burden of liver and biliary diseases caused by *C. sinensis* infection in Guangdong Province amounts to 1.6 billion Chinese yuan (CNY) [5]. This economic burden includes the direct economic burden of the disease, direct non-medical costs, and indirect economic burden, which cover expenditures from medical insurance, out-of-pocket payments, public funding, and other sources.

Clonorchiasis is a food-borne parasitic disease primarily transmitted through the ingestion of raw or undercooked freshwater fish that contain encysted metacercariae of *C. sinensis* [4, 6–8]. Following infection, most of the individuals remain asymptomatic or experience mild symptoms, while some turn into substantial clinical or subclinical liver and biliary diseases, with cholangiocarcinoma (CCA) being the severe and fatal complication [9]. In 2009, *C. sinensis* was classified as a definite human carcinogenic (Group 1) by the International Agency for Research on Cancer (IARC) [10, 11]. Currently, the main treatment for clonorchiasis is chemotherapy [12]. Considering the high economic burden caused by symptomatic therapy for sequelae, the prevention strategy, which includes preventive and curative measures, was proposed [13, 14].

According to conservative estimation, approximately 15 million people worldwide have been infected with *C. sinensis*, with 85% of the cases attributed to China [15]. However, the degree of clonorchiasis endemicity in China varies across regions, with higher concentrations in the southeast and northeast [16]. Guangdong Province was found to be one of the most severely infected provinces according to the three large-scale nationwide surveys conducted in 1990, 2003, and 2015 [16, 17]. The natural conditions, such as abundant river bodies and a climate suitable for the parasite, combined with the local dietary culture of eating raw fish, have contributed to the endemicity of clonorchiasis in Guangdong. The prevalence of the disease has been steadily increasing in Guangdong since 1990 [16, 17]. In response, the Guangdong government issued the Prevention and Control Program for Key Parasitic Diseases (2016–2020) in 2016, implementing a series of prevention strategies were adopted [18]. It was assumed that high infection intensity and number of re-infections among hosts in endemic areas were associated with chronic infection, which might result in more severe health problems and produce a higher disease burden. Thus, it is of great importance to evaluate the current disease burden caused by clonorchiasis and investigate its temporal trends and distribution characteristics in Guangdong to facilitate the development of control strategies.

As defined by the World Health Organization (WHO), the disease burden was not only limited to incidence, mortality, and disability-adjusted life years (DALYs) [19] but also included the economic burden. Several studies have investigated the disease burden of clonorchiasis, among which most researchers followed the Global Burden of Disease (GBD) study and selected disability-adjusted life years as the measurement, and only one study evaluated the economic burden measured by currency. Furst et al. [20] found that the global disease burden caused by clonorchiasis in 2005 was approximately 275,370 DALYs, with China accounting for 84.1% (231,547 DALYs) and Vietnam, South Korea and Russia accounting for 15.9% (43,823 DALYs). Another study reported a higher figure with the DALYs owing to clonorchiasis were 489,174.04 person-years in China [21]. As for the economic burden, it was calculated that 1.3 billion CNY was created by *C. sinensis* infection in Guangdong [5]. Although research has been carried out on the estimation of the disease burden of clonorchiasis, only a few studies focused on endemic regions, and there was a lack of studies using comprehensive indicators.

Therefore, the primary objective of this study is to evaluate the current disease burden of clonorchiasis in Guangdong Province by utilizing synthesized indexes such as prevalence, DALYs, and cost of illness. Furthermore, the study aims to analyze the trends and distribution characteristics after the implementation of an integrated preventive strategy.

## Methods

### Study areas

Guangdong was one of the seven high clonorchiasis prevalence settings and was chosen to be the disease surveillance province [22]. Thus, an annual survey in fixed monitoring stations and mobile monitoring stations was carried out to investigate the current epidemic status of clonorchiasis from 2016 to 2021. The study included 16 fixed national surveillance sites, which were categorized according to their prevalence rate into low prevalence regions (≤ 10%), middle prevalence regions (> 10% and ≤ 30%), and high prevalence regions (> 30%). In each region, five villages or communities were selected from five different directions, including north, south, east, west, and middle. Subsequently, 200 villagers or citizens aged > 3 years were included in the survey from each unit. The study used a stratified random sampling method to select participants from each of the 16 fixed national surveillance sites. The sample size was determined based on the estimated prevalence rate of clonorchiasis in each region. The same sampling method was adopted for mobile surveillance sites.

### Data collection

The prevalence survey data from 2016 to 2021 was collected from the Guangdong Provincial Center for Disease Control and Prevention, using methods such as reporting cases and active disease surveillance. The annual survey included fixed monitoring stations and mobile monitoring stations, which were used to collect data from the sampled individuals. Guangdong Province has established six national monitoring sites for liver fluke, two national comprehensive prevention and control demonstration areas, and eight provincial mobile monitoring sites. Among them, there are three fixed national monitoring sites, each with an annual budget of 45,000 CNY (6690.2 United States dollars, USD), totaling 135,000 CNY (20,070.5 USD); three mobile national monitoring sites, each with an annual budget of 50,000 CNY (7,433.5 USD), totaling 150,000 CNY (22,300.5 USD); two comprehensive prevention and control project counties, each with an annual budget of 150,000 CNY (22,300.5 USD), totaling 300,000 CNY (44,601.0 USD); and eight provincial monitoring sites, each with an annual budget of 50,000 CNY (7433.5 USD), totaling 400,000 CNY (59,468.1 USD). The total budget amounts to 985,000 CNY (146,440.1 USD). In addition, the study also utilized data from community health centers, hospitals, and clinics to ensure a comprehensive collection of prevalence data for clonorchiasis.

The sample size for the infection rate is specifically based on the 'Monitoring Plan for Clonorchiasis and Soil-Transmitted Helminthiases in Guangdong Province (Trial).' This study employed the modified Kato-Katz thick smear technique to examine and report egg counts. According to infection intensity classifications, > 98% of the infected individuals in the annual monitoring data had light infections (< 1000 eggs per gram of feces). The data included information on the number of examined persons, the number of persons infected with *C. sinensis*, and the general demographic information (e.g. age, sex, occupation, and education) of each examined individual. The crucial epidemiological disease parameters and demographic information, which were used to calculate DALYs, were obtained from literature and Guangdong Statistic Yearbooks or official documents. The cost data for treating clonorchiasis were acquired from a tertiary hospital in Guangdong Province, which was selected based on its reputation as a leading provider of healthcare services in the region. The hospital is known for its expertise in the treatment of infectious diseases, including clonorchiasis. While the hospital may not be representative of all healthcare facilities in Guangdong Province, it was chosen due to its status as a tertiary hospital and its extensive experience in treating clonorchiasis. The direct medical cost data were sourced from the medical information system of the hospital. Using International Classification of Diseases ten version (ICD-10) coding, the names of major diagnosed diseases between 2008 and 2022 (Clonorchiasis, Clonorchiasis (Chinese liver fluke disease), Clonorchiasis infection, parasitic infection, trematode infection, and Chinese liver fluke disease) were identified. Sociodemographic information (e.g. age, sex, ethnic and marital status), cost information, and healthcare utilization (length of stay) information of each case was included in the database.

A total of 124 patient medical records were selected for analysis. The costs were adjusted to 2022 CNY levels using the consumer price index, and extreme values (*n* = 114) were excluded before conducting the calculations. Considerations for the inclusion criteria of certain studies were as follows The *C. sinensis* infection group consisted of patients discharged with diagnoses of *C. sinensis* infection complicated by obstructive jaundice, cholecystitis, cholangitis, gallstones, cirrhosis, or malignant liver tumors. The non-*C. sinensis* infection group comprised patients discharged without diagnoses of *C. sinensis* infection but with complications such as obstructive jaundice, cholecystitis, cholangitis, gallstones, cirrhosis, or malignant liver tumors. Therefore, in this study, we considered the secondary disease burdens of obstructive jaundice, cholecystitis, cholangitis, gallstones, cirrhosis, and malignant liver tumors resulting from *C. sinensis* infection. Consequently, only patients with uncomplicated *C. sinensis* infections were included in the analysis for this study, excluding any cases with complications arising from the infection.

### Data analysis

#### Calculation of DALYs

DALY is an index used to quantify health loss caused by a disease, and one DALY can be considered as one lost year of healthy life due to morbidity [22–25]. There are two methods for the calculation of DALY. One is based on incidence, and the other is based on prevalence [25]. DALY is calculated by adding years of life lost (YLLs) due to premature death from disease and years lived with disability (YLDs) owing to morbidity [25]. The formula was as follows:

$$DALYs=YLDs+YLLs$$

In this study, the calculation of DALYs used a prevalence approach. YLDs were calculated by multiplying the number of the infected (number of population*prevalence rate of clonorchiasis) with corresponding disability weights (DW). The disability weights range from zero to one, and one represents the most severe health outcome of death. The specific calculation process is listed in formula (a).

The calculation of YLLs followed the method adopted by previous studies [20, 21]. Premature death from *C. sinensis* was thought to be the attributable case of CCA. The odds ratio between *C. sinensis* infection and CCA and the incidence of CCA was used to compute the incidence rate of CCA, which was attributable to *C. sinensis* (I*(*OR*-1)). Considering the poor prognosis and short disease duration of CCA [12], the mortality rate was replaced by its incidence rate. Finally, YLLs were calculated by the summation of all fatal populations multiplied by the life span at the age of death (*L*_*e*_-*L*_*d*_). The detailed procedure is expressed in formula (b).

The parameters used for evaluation were listed as follows. An estimation of the infection rate was obtained by dividing the number of infected by the number of examined persons. The provincial and municipal population was derived from the Guangdong Statistic Yearbooks [26]. The weighted disability weight due to *C. sinensis* in the Chinese population was 0.075 [95% confidential interval (95% CI): (0.060, 0.091)] [9]. The incidence rate of CCA among patients infected with *C. sinensis* was 1.5 per 100,000 population [27], and the odds ratio between *C. sinensis* and CCA was 4.47 [95% CI (2.61, 7.66)] [15]. The average life expectancy of the Guangdong population was 79.6 years old. As for the age of death in patients with CCA, it was replaced by the average age at the time of diagnosis. The figure used in the evaluation was 62.6 [95% CI (62.4, 62.8)] years old [28].

#### Calculation of cost of illness

The electronic medical records provided data for calculating direct medical costs from the healthcare system’s perspective. The direct calculation approach was used to evaluate the direct medical costs caused by clonorchiasis. In terms of composition regulated by the government, direct medical costs were made up of bed fees, diagnostic fees, treatment fees, and other fees. Considering the infection characteristics and high cure rate of clonorchiasis [29], the direct medical costs per case were calculated based on each record. Patients with re-infection were counted as multiple cases. Considering inflation, costs were adjusted to 2021 CNY value using the Consumer Price Index (CPI).

The prevalence rate of *C. sinensis* infection was calculated for each municipality in Guangdong Province, with the definition of positive infection being the presence of at least one egg. The 95% *CI* was calculated using a one-sample t-test. Comparisons of prevalence rates among people with different characteristics were achieved through the chi-square test. The significance level was set at 0.05. The main examined variables, DALYs and DALYs per 1000 population, were calculated by definition issued by WHO. The propagating imprecision approach was used to produce a 95% CI of DALYs [30]. The spatial distributions of DALYs and DALYs per 1000 population were demonstrated through maps, while the temporal trends were illustrated by line chart. As for cost data, descriptive statistics, including mean, standard deviation, median, percentiles, and proportion, were used to depict sociodemographic characteristics and direct medical costs. Due to the skewed distribution of medical costs, comparisons of costs for groups with different characteristics were made through the rank sum test (Mann-Whitney U test for two groups and Kruskal-Wallis H test for three and above groups). Statistical analyses were performed with Microsoft Excel 2019 ((Microsoft, Albuquerque, NM, USA) and R language (version 4.2.2). *P < 0.05 is considered statistically significant.

## Results

A total of 129,582 people were examined to determine the infection level of *C. sinensis* in Guangdong Province from 2016 to 2021, with 10,270 in 2016, 17,332 in 2017, 21,577 in 2018, 22,879 in 2019, 28,880 in 2020, and 28,644 in 2021. The total number of infections was 4210, and the annual number of infections from 2016 to 2021 was 773, 856, 692, 417, 254, and 1218, respectively. The overall prevalence rate of clonorchiasis was 3.25% [95% CI 3.15%, 3.35%].

The infection rate of *C. sinensis* in Guangdong Province showed a decreasing trend between 2016 and 2020 and then staged a recovery in 2021. The infection rates from 2016 to 2021 were 7.53 [95% CI (7.02, 8.05)], 4.94 [95% CI (4.62, 5.27)], 3.21 [95% CI (2.98, 3.45)], 1.82 [95% CI (1.65, 2.00), 0.88 [95% CI (0.78, 0.99)], and 4.25 [95% CI (4.02, 4.49)], respectively (Table 1). The chi-square test indicated that the difference in infection rates in different years was remarkable (*χ*^2^ = 1511.1, *P* < 0.001).

**Table 1:** Infection rate (%) of *Clonorchis sinensis* in Guangdong Province and its prefecture-level cities

City	In 2016	In 2017	In 2018	In 2019	In 2020	In 2021
Chaozhou	/	0.00 (0.00, 0.37)	0.00 (0.00, 0.37)	0.00 (0.00, 0.37)	/	0.00 (0.00, 0.37)
Dongguan	0.00 (0.00, 0.37)	/	/	/	0.00 (0.00, 0.32)	0.00 (0.00, 0.32)
Foshan	7.33 (5.80, 9.12)	17.25 (14.96, 19.73)	34.77 (31.91, 37.71)	8.57 (6.92, 10.48)	1.73 (1.03, 2.71)	11.13 (9.26, 13.23)
Guangzhou	12.23 (10.27, 14.40)	3.04 (2.06, 4.31)	0.00 (0.00, 0.37)	0.00 (0.00, 0.36)	0.10 (0.00, 0.56)	2.90 (1.95, 4.14)
Heyuan	/	30.40 (27.56, 33.36)	0.25 (0.05, 0.72)	0.32 (0.13, 0.66)	0.79 (0.45, 1.28)	37.15 (34.57, 39.79)
Huizhou	0.13 (0.00, 0.70)	0.31 (0.06, 0.92)	0.00 (0.00, 0.37)	0.10 (0.00, 0.55)	0.30 (0.11, 0.65)	0.00 (0.00, 0.36)
Jiangmen	23.10 (20.55, 25.80)	15.52 (13.34, 17.91)	13.03 (11.02, 15.25)	6.94 (5.88, 8.13)	7.74 (6.18, 9.54)	12.83 (11.65, 14.07)
Jieyang	/	0.00 (0.00, 0.37)	0.09 (0.00, 0.50)	0.00 (0.00, 0.36)	0.00 (0.00, 0.35)	0.00 (0.00, 0.37)
Maoming	/	0.00 (0.00, 0.37)	0.10 (0.00, 0.54)	0.00 (0.00, 0.37)	0.00 (0.00, 0.37)	0.00 (0.00, 0.37)
Meizhou	/	10.07 (8.35, 12.01)	0.00 (0.00, 0.37)	0.00 (0.00, 0.36)	7.01 (5.51, 8.78)	0.00 (0.00, 0.37)
Qingyuan	0.35 (0.10, 0.90)	2.38 (1.56, 3.47)	2.99 (2.03, 4.25)	0.79 (0.34, 1.54)	0.58 (0.21, 1.25)	0.19 (0.02, 0.67)
Shantou	/	0.00 (0.00, 0.37)	0.00 (0.00, 0.37)	0.00 (0.00, 0.36)	0.00 (0.00, 0.36)	0.00 (0.00, 0.36)
Shanwei	/	/	0.00 (0.00, 0.37)	0.00 (0.00, 0.37)	0.00 (0.00, 0.37)	0.00 (0.00, 0.37)
Shaoguan	0.18 (0.02, 0.64)	0.53 (0.27, 0.95)	0.22 (0.07, 0.50)	0.04 (0.00, 0.25)	0.00 (0.00, 0.16)	0.38 (0.16, 0.74)
Shenzhen	0.21 (0.03, 0.76)	0.30 (0.06, 0.87)	0.89 (0.41, 1.68)	0.88 (0.40, 1.66)	0.50 (0.24, 0.91)	1.08 (0.71, 1.58)
Yangjiang	/	(0.00, 0.00)	0.00 (0.00, 0.37)	0.00 (0.00, 0.37)	0.00 (0.00, 0.18)	0.00 (0.00, 0.30)
Yunfu	/	2.40 (1.54, 3.55)	0.10 (0.00, 0.57)	0.00 (0.00, 0.34)	0.09 (0.00, 0.50)	0.38 (0.10, 0.97)
Zhanjiang	0.00 (0.00, 0.36)	0.00 (0.00, 0.35)	0.00 (0.00, 0.34)	0.00 (0.00, 0.33)	0.00 (0.00, 0.34)	0.00 (0.00, 0.36)
Zhaoqing	/	/	6.55 (5.10, 8.26)	/	0.37 (0.21, 0.61)	1.28 (0.91, 1.75)
Zhongshan	27.43 (24.9230.06)	/	8.91 (6.92, 11.26)	8.08 (6.49, 9.90)	2.89 (1.95, 4.13)	9.33 (7.62, 11.29)
Zhuhai	/	1.28 (0.70, 2.14)	0.89 (0.41, 1.68)	7.64 (6.10, 9.43)	0.20 (0.02, 0.72)	1.06 (0.53, 1.88)
Guangdong	7.53 (7.02, 8.05)	4.94 (4.62, 5.27)	3.21 (2.98, 3.45)	1.82 (1.65, 2.00)	0.88 (0.78, 0.99)	4.25 (4.02, 4.49)

As for spatial distribution, *C. sinensis* infection appeared in area clustering in Guangdong Province, concentrating in the Pearl River and Han River basins. Of 21 prefecture-level cities, Jiangmen, Foshan, Zhongshan, and Heyuan had the highest infection rates. In 2021, the figures respectively reached 12.8%, 11.1%, 9.3%, and 37.2%. The prevalence situation was similar to that in other years (Table 1).

In terms of population distribution, people of different sex, age, occupation, and education showed significant differences in prevalence rates. Table 2 shows the infection rate of *C. sinensis*, which was stratified according to sex, age group, occupation, education, and years. Men had a significantly higher prevalence rate (4.27%) than women (2.26%) for infections with *C. sinensis* (*χ*^2^ = 413.3, *P* < 0.001). People aged < 18 years old were less likely to have *C. sinensis* infection (0.54%) compared with individuals in other age groups (*χ*^2^ = 1860.1, *P* < 0.001). With increase in age, the prevalence rose at the beginning but then it decreased, and the prevalence peak appeared in the 41–50-year-old group. Students had the lowest prevalence rate (0.69%) among all occupations (*χ*^2^ = 1041.0, *P* < 0.001). The *C. sinensis* infection rate was the highest in people with a college degree (5.21%), followed by those with high school degrees (5.18%), illiteracy (4.63%), junior school degrees (3.36%), and elementary school education (1.80%). A significant difference was observed in people with different education levels (*χ*^2^ = 818.4, *P* < 0.001).

**Table 2:** Population distribution of *Clonorchis sinensis* infection rate in Guangdong Province

Characteristics	2016		2017		2018		2019		2020		2021		Total	
	Examined population	Infections (infection rate %)	Examined population	Infections (infection rate %)	Examined population	Infections (infection rate %)	Examined population	Infections (infection rate %)	Examined population	Infections (infection rate %)	Examined population	Infections (infection rate %)	Examined population	Infections (infection rate%)
Sex														
Male	4924	468 (9.50)	8701	589 (6.77)	10,774	407 (3.78)	11,159	252 (2.26)	13,869	163 (1.18)	14,190	837 (5.90)	63,617	2716 (4.27)
Female	5346	305 (5.71)	8631	267 (3.09)	10,803	285 (2.64)	11,720	165 (1.41)	15,011	91 (0.61)	14,454	381 (2.64)	65,965	1494 (2.26)
Age groups														
< 18	3119	67 (2.15)	5644	41 (0.73)	7139	26 (0.36)	8525	31 (0.36)	10,106	11 (0.11)	8622	57 (0.66)	43,155	233 (0.54)
18–30	1239	73 (5.89)	1728	67 (3.88)	2483	59 (2.38)	2582	49 (1.90)	2654	24 (0.90)	2651	101 (3.81)	13,337	373 (2.80)
31–40	1610	168 (10.43)	2813	136 (4.83)	3496	145 (4.15)	3173	95 (2.99)	4198	40 (0.95)	5343	249 (4.66)	20,633	833 (4.04)
41–50	1511	138 (9.13)	2736	218 (7.97)	2870	135 (4.70)	2821	86 (3.05)	3352	52 (1.55)	3830	376 (9.82)	17,120	1005 (5.87)
51–60	1397	167 (11.95)	2097	222 (10.59)	2545	131 (5.15)	2545	84 (3.30)	3743	58 (1.55)	3717	272 (7.32)	16,044	934 (5.82)
61–70	932	123 (13.20)	1471	120 (8.16)	2040	147 (7.21)	2175	54 (2.48)	3085	42 (1.36)	2901	112 (3.86)	12,604	598 (4.74)
> 70	461	37 (8.03)	842	52 (6.18)	1004	49 (4.88)	1058	18 (1.70)	1742	27 (1.55)	1580	51 (3.23)	6687	234 (3.50)
Occupations														
Student	1824	68 (3.73)	4261	40 (0.94)	4620	15 (0.32)	6355	37 (0.58)	7265	8 (0.11)	5647	40 (0.71)	29,972	208 (0.69)
Worker	1306	91 (6.97)	1481	67 (4.52)	2528	202 (7.99)	2004	73 (3.64)	2855	57 (2.00)	2602	129 (4.96)	12,776	619 (4.85)
Peasant	3055	380 (12.44)	5963	295 (4.95)	6288	193 (3.07)	8188	206 (2.52)	9175	110 (1.20)	6491	263 (4.05)	39,160	1447 (3.70)
Public official	835	34 (4.07)	441	49 (11.11)	840	80 (9.52)	668	45 (6.74)	1437	19 (1.32)	2398	173 (7.21)	6619	400 (6.04)
Teacher and medical staff	380	31 (8.16)	750	71 (9.47)	878	26 (2.96)	727	14 (1.93)	1113	13 (1.17)	1494	151 (10.11)	5342	306 (5.73)
Other	2870	169 (5.89)	4436	334 (7.53)	6423	176 (2.74)	4937	42 (0.85)	7035	47 (0.67)	10,012	462 (4.61)	35,713	1230 (3.44)
Education														
Illiterate and semiliterate	425	31 (7.29)	634	87 (13.72)	596	16 (2.68)	660	9 (1.36)	531	7 (1.32)	721	15 (2.08)	3567	165 (4.63)
Preschool and primary school	4161	300 (7.21)	7308	191 (2.61)	9028	196 (2.17)	9745	84 (0.86)	12,803	63 (0.49)	11,460	148 (1.29)	54,505	982 (1.80)
Junior	2466	167 (6.77)	4630	257 (5.55)	6233	178 (2.86)	6789	149 (2.19)	8486	90 (1.06)	6879	352 (5.12)	35,483	1193 (3.36)
Senior	1714	141 (8.23)	3271	223 (6.82)	3760	177 (4.71)	3816	122 (3.25)	3950	67 (1.70)	5398	404 (7.48)	21,909	1134 (5.18)
College	1504	134 (8.91)	1489	98 (6.58)	1960	125 (6.38)	1867	53 (2.84)	3110	27 (0.87)	4186	299 (7.14)	14,116	736 (5.21)

The overall disease burden resulting from clonorchiasis in Guangdong was 406,802.29 [95% CI (329,275.33, 4,9215,163.78)] person-years in 2021, consisting of 402,390.00 [95% *CI* (324,984.35, 4,8761,687.27)] YLDs and 4,412.29 [95% CI (2,046.96, 847,007.02)] YLLs. The DALYs per 1000 population were 3.22 [95% CI (2.61, 389.85)] person-years. Among the 21 prefecture-level cities in Guangdong Province, Foshan, Heyuan, and Zhongshan showed the highest disease burden measured by DALYs (Table 3). The spatial distribution of DALYs caused by *C. sinensis* infection in each municipality across Guangdong is displayed in Fig. 1.

**Table 3:** DALY and its 95% confidential interval of clonorchiasis in Guangdong Province in 2021

City	Infection rate (%)	No. population in 2020 (person)	YLLs (person-years)	YLDs (person-years)	DALYs (person-years)	DALYs per 1000 population (person-year/1000 persons))
Chaozhou	0.00	2,566,600	0.00 (0.00, 315.25)	0.00 (0.00, 28,334.49)	0.00 (0.00, 28,595.20)	0.00 (0.00, 11.14)
Dongguan	0.00	10,483,600	0.00 (0.00, 1121.94)	0.00 (0.00, 100,839.09)	0.00 (0.00, 101,766.91)	0.00 (0.00, 9.71)
Foshan	11.13	9,518,800	871.27 (403.72, 167,741.56)	79,458.18 (61,689.57, 10,050,717.92)	80,329.46 (62,505.74, 10,143,964.25)	8.44 (6.57, 1065.68)
Guangzhou	2.90	18,740,300	446.94 (206.02, 87,081.77)	40,760.15 (29,839.38, 5,543,173.73)	41,207.10 (30,235.02, 5,594,478.08)	2.20 (1.61, 298.53)
Heyuan	37.15	2,835,600	866.33 (401.86, 166,323.72)	79,006.91 (63,472.16, 9,619,644.69)	79,873.23 (64,310.47, 9,709,076.54)	28.17 (22.68, 3423.99)
Huizhou	0.00	6,057,200	0.00 (0.00, 726.59)	0.00 (0.00, 65,305.22)	0.00 (0.00, 65,906.09)	0.00 (0.00, 10.88)
Jiangmen	12.83	4,804,100	506.89 (235.03, 97,325.58)	46,227.45 (36,848.72, 5,675,174.73)	46,734.35 (37,335.60, 5,727,907.02)	9.73 (7.77, 1192.30)
Jieyang	0.00	5,578,700	0.00 (0.00, 683.18)	0.00 (0.00, 61,403.30)	0.00 (0.00, 61,968.27)	0.00 (0.00, 11.11)
Maoming	0.00	6,180,000	0.00 (0.00, 759.08)	0.00 (0.00, 68,225.34)	0.00 (0.00, 68,853.08)	0.00 (0.00, 11.14)
Meizhou	0.00	3,871,000	0.00 (0.00, 475.47)	0.00 (0.00, 42,734.67)	0.00 (0.00, 43,127.88)	0.00 (0.00, 11.14)
Qingyuan	0.19	3,974,000	6.21 (2.68, 1572.90)	566.30 (321.68, 123,773.24)	572.50 (325.97, 124,913.92)	0.14 (0.08, 31.43)
Shantou	0.00	5,503,700	0.00 (0.00, 652.56)	0.00 (0.00, 58,651.63)	0.00 (0.00, 59,191.28)	0.00 (0.00, 10.75)
Shanwei	0.00	2,669,400	0.00 (0.00, 327.88)	0.00 (0.00, 29,469.37)	0.00 (0.00, 29,740.52)	0.00 (0.00, 11.14)
Shaoguan	0.38	2,855,300	8.92 (4.01, 1812.40)	813.76 (535.07, 126,677.38)	822.68 (542.18, 127,847.11)	0.29 (0.19, 44.78)
Shenzhen	1.08	17,633,800	156.62 (72.00, 30,533.13)	14,283.38 (10,364.43, 1,958,427.39)	14,440.00 (10,501.88, 1,976,549.15)	0.82 (0.60, 112.09)
Yangjiang	0.00	2,605,900	0.00 (0.00, 259.68)	0.00 (0.00, 23,340.12)	0.00 (0.00, 23,554.88)	0.00 (0.00, 9.04)
Yunfu	0.38	2,383,700	7.45 (3.34, 1656.26)	679.35 (422.06, 122,919.14)	686.80 (427.68, 124,052.95)	0.29 (0.18, 52.04)
Zhanjiang	0.00	6,980,700	0.00 (0.00, 829.28)	0.00 (0.00, 74,535.29)	0.00 (0.00, 75,221.09)	0.00 (0.00, 10.78)
Zhaoqing	1.28	4,116,900	43.34 (20.03, 8417.06)	3952.22 (2939.46, 527,988.12)	3995.56 (2978.41, 532,877.25)	0.97 (0.72, 129.44)
Zhongshan	9.33	4,431,100	339.99 (157.48, 65,499.81)	31,006.62 (23,934.33, 3,947,990.51)	31,346.62 (24,251.07, 3,984,607.79)	7.07 (5.47, 899.24)
Zhuhai	1.06	2,449,600	21.35 (9.73, 4287.72)	1947.43 (1329.96, 291,821.89)	1968.79 (1347.62, 294,518.04)	0.80 (0.55, 120.23)
Guangdong	4.25	126,240,000	4412.29 (2046.96, 847,007.02)	402,390.00 (324,984.35, 48,761,687.27)	406,802.29 (329,275.33, 49,215,163.78)	3.22 (2.61, 389.85)

**﻿Fig. 1:**
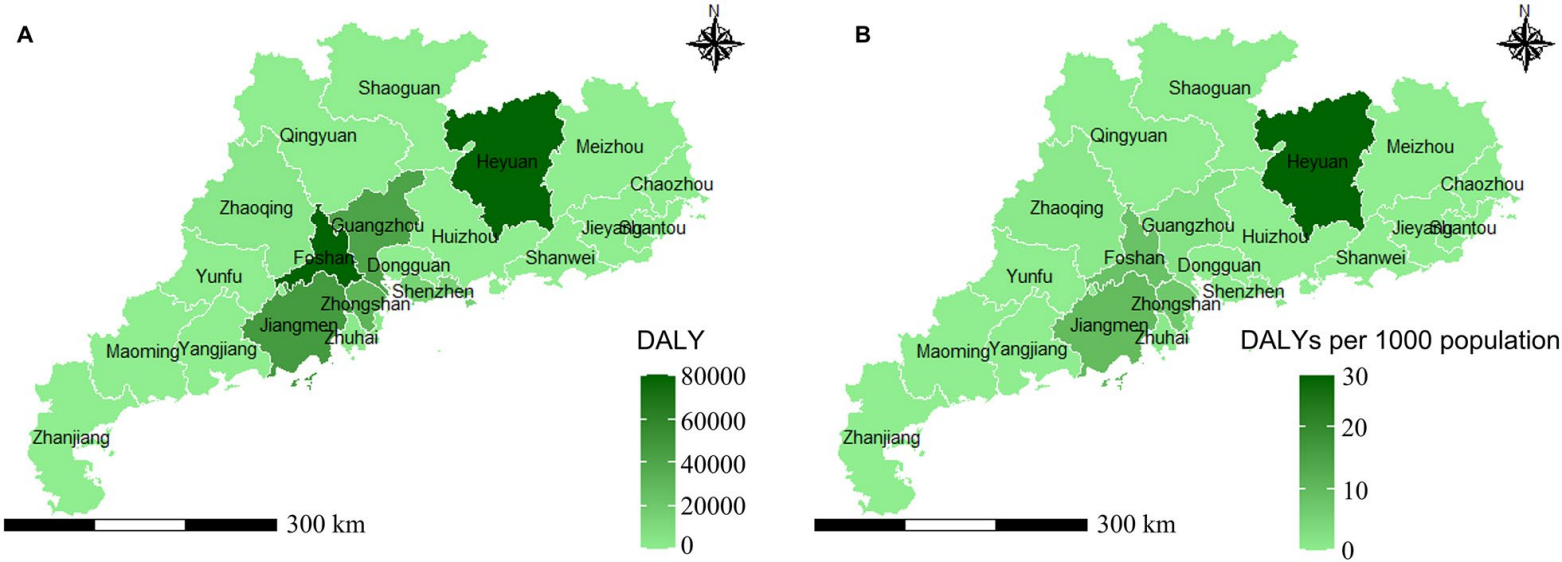
Distribution of the disease burden of clonorchiasis in Guangdong Province in 2021. **A** DALY. **B** DALY per 1000 persons

**Fig. 2:**
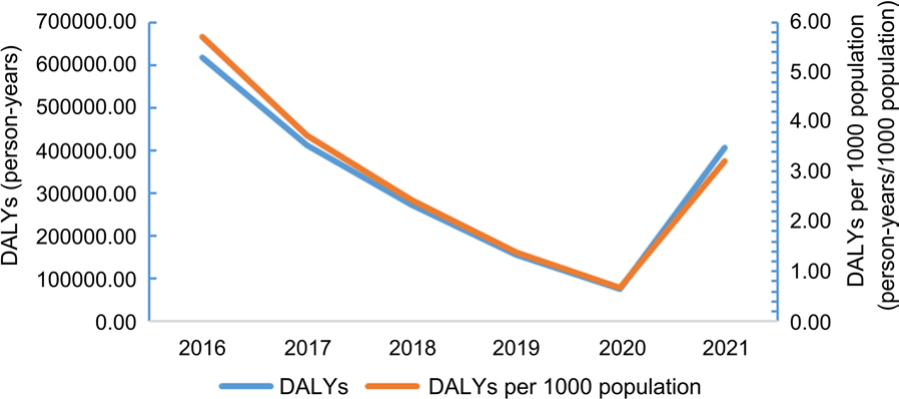
Temporal trends of DALYs of clonorchiasis in Guangdong Province

From 2016 to 2021, the disease burden indicators, including DALYs and DALYs per 1000, gradually decreased at the beginning of 5 years and then rose suddenly (Fig. 2), consistent with the temporal trends of prevalence rate. However, the overall trends of DALYs and DALYs per 1000 presented a downward trend, decreasing from 618,862.85 and 5.70 in 2016 to 406,802.29 and 3.22 in 2021 (Table 4).

**Table 4:** DALYs of clonorchiasis in Guangdong Province in 2016–2021

Year	Infection rate (%)	No. population in 2020 (person)	YLL (person-year)	YLD (person-year)	DALY (person-year)	DALY per 1000 population (person-year/1000 persons))
2016	7.53	108,490,000	6165.57	612,697.28	618,862.85	5.70
2017	4.94	109,990,000	4129.08	407,512.95	411,642.03	3.74
2018	3.21	111,690,000	2724.54	268,893.68	271,618.21	2.43
2019	1.82	113,460,000	1569.23	154,872.90	156,442.13	1.38
2020	0.88	115,210,000	802.12	76,038.60	76,840.72	0.67
2021	4.25	126,240,000	4412.29	402,390.00	406,802.29	3.22

In total, 124 patients with the primary diagnosis of clonorchiasis were identified. After excluding extreme values, 114 patients were included for cost analysis. The basic characteristics are displayed in Table 5. Out of the total cases, males accounted for 80.7%. The mean age was 44.9 (*SD* = 12.0) years old. Among all age groups, more cases were classified into the age group 40 to 60 years (47.4%), followed by the group < 40 years (37.7%) and > 60 years (14.9%). Of all the ethnic groups, the Han patients comprised the majority, with a proportion of 99.1%. As for marital status, 87.7% of patients were married. The average length of stay was 8.4 (*SD* = 4.4) days.

**Table 5:** Demographic characteristics of patients with clonorchiasis

Characteristics	Total sample (*n* = 114)
Sex	
Female, *n* (%)	22.0 (19.3)
Male, *n* (%)	92.0 (80.7)
Age	
Mean ± sd	44.9 ± 12.0
Median (25th–75th)	43.0 (36.0–54.0)
Age groups	
< 40, *n* (%)	43.0 (37.7)
40–60, *n* (%)	54.0 (47.4)
> 60, *n* (%)	17.0 (14.9)
Ethnic group	
Han, *n* (%)	113.0 (99.1)
Other, *n* (%)	1.0 (0.9)
Marital status	
Married, *n* (%)	100.0 (87.7)
Other, *n* (%)	14.0 (12.3)
Length of stay	
Mean ± sd	8.4 ± 4.4
Median (25th–75th)	7.0 (5.0–11.0)
< 8 days	62 (54.4)
≥ 8 days	52 (45.6)

Overall, the direct medical costs of patients with clonorchiasis per case were CNY 7907.2 (*SD* = 5154.4) (equivalent to 1175.7 USD ± 766.3) (Table 6). Costs did not show significant differences among patients with different sex, age, ethnicity, and marital status, while the costs for patients having different lengths of stay were significantly different (*P* < 0.001).

**Table 6:** Direct medical costs of clonorchiasis for patients with different characteristics

Characteristics	Direct medical costs (CNY) Mean ± SD	Direct medical costs (USD^a^) Mean ± SD	*P* value
Direct medical costs	7907.2 ± 5154.4	1175.7 ± 766.3	
Sex			0.085
Female	6960.9 ± 5951.8	1.34.9 ± 88.49	
Male	8133.5 ± 4954.6	1209.2 ± 736.6	
Age groups (years)			0.980
< 40	7916.5 ± 5115.6	1176.9 ± 760.5	
40–60	7688.6 ± 4690.1	1143.1 ± 697.3	
> 60	8578.0 ± 6740.8	1275.3 ± 1002.2	
Ethnic group			0.202
Han	7845.9 ± 5135.5	1166.5 ± 763.5	
Other	/		
Marital status			0.839
Married	7983.1 ± 5229.1	1186.8 ± 777.4	
Other	7365.2 ± 4727.0	1095.0 ± 702.8	
Length of stay (days)			< 0.001*
< 8 days	5033.4 ± 2987.0	748.3 ± 444.1	
≥ 8 days	11,333.7 ± 5117.0	1685.0 ± 760.7	

^*^Statistically significant

^a^Based on the average exchange rate of 1 USD to 6.7263 CNY in 2022

## Discussion

In Guangdong, *C. sinensis* infections are highly prevalent, and the occurrence of repeated and chronic infection is common [13, 31, 32]. Notably, few studies have previously explored the disease burden of clonorchiasis in endemic areas using synthesized indexes. This study aimed to evaluate the disease burden caused by *C. sinensis* infection through comprehensive indicators and assess its trends from 2016 to 2021 in Guangdong.

It was found that the burden of disease associated with clonorchiasis in Guangdong was overwhelming, with a prevalence rate of 4.25%, DALYs of 406,802.29 [95% CI (329,275.33, 49,215,163.78)] person-years, and direct medical costs of CNY 7907.2 (USD 1175.7) in 2021. The prevalence rate, DALYs, and DALYs per 1000 population all experienced a downward trend between 2016 and 2020, then an upward trend in 2021, while in general, the situation in 2021 was better than in 2016. Moreover, the distribution of disease burden also showed spatial clustering, with infection cases concentrated in the Pearl River and Han River basins, such as Foshan, Zhongshan, and Heyuan. As for population distribution, people with different characteristics tend to have different prevalence rates and direct medical costs.

Based on the 2021 provincial clonorchiasis surveillance survey results, the prevalence rate of *C. sinensis* infection was 4.25% [95% CI (4.02, 4.49)]. The DALYs and DALYs per 1000 population due to clonorchiasis were 406,802.29 [95% CI (329,275.33, 49,215,163.78)] person-years and 3.22 [95% CI (2.61, 389.85)], respectively. Zhao et al. [20] found that the burden of clonorchiasis in Guangdong was 157,245.48 [95% CI (120,532.89, 199,807.40)] DALYs in 2015, and the global burden of clonorchiasis reported by Furst et al. [20] was 231,547 person-years. Compared with previous research, this study reported a much higher figure. The different computation methods and study time might explain the condition [21]. In the former study, a standardized prevalence rate was used for calculation, and thus the results might not accurately reflect the reality, while in the latter study, only severe disability due to infection was considered, which might lead to the result of lower disease burden. Different from these two studies, this research considered all severity levels of disability due to *C. sinensis* infection and adopted the latest raw prevalence rate for calculation. Therefore, to some extent, the results in this study could precisely reflect the current disease burden caused by clonorchiasis in Guangdong.

Through cost analysis of clonorchiasis, we found that the average direct medical costs per case in Guangdong were CNY 7,907.2 (*SD* = 5154.4) (equivalent to 1175.7 USD ± 766.3), lower than the previous research findings [5]. In that study, the calculated direct medical costs of clonorchiasis-induced gallbladder cholangitis, cholelithiasis, liver cirrhosis, and malignant liver tumor were respectively CNY 5015.52, CNY 9971.76, CNY 13,168.83, and CNY 17,638.28 in 2009 (CNY 7034.32, CNY 13,985.50, CNY 18,469.42, and CNY 24,737.87 in 2022) [33]. Different selection criteria for the study population might partly explain the situation. In this study, patients whose primary diagnosis was clonorchiasis were chosen, and the symptoms were relatively mild, while in that study, patients with liver and biliary disease were selected. Therefore, the costs in our study were relatively lower and closer to the real world’s true values.

From 2016 to 2021, the prevalence rate and DALYs of clonorchiasis decreased steadily first and then increased in 2021. The continued decline in the first 5 years might be attributed to the implementation of the Clonorchiasis Prevention and Control Project in Guangdong Province. Since 2016, the relevant departments have taken a series of measures such as propaganda and education, surveillance, and deworming treatment to prevent clonorchiasis [18]. Thus, the main source of contagion and transmission route were controlled, and the prevalence rate and DALYs decreased correspondingly. For the rise in 2021, one possible explanation was that, owing to the pandemic of COVID-19, there was a lack of human, physical, and financial resources. It was indicated that the shortage of resources was an obstacle to the prevention of clonorchiasis [34]. Therefore, the prevalence rate and DALYs showed a slight rebound in 2021.

The results revealed that the clonorchiasis cases were distributed in most areas of Guangdong Province, with the prevalence rate and DALYs primarily located alongside the Pearl River and Han River, consistent with the results of previous prevalence surveys conducted in Guangdong [35–37]. There were several reasons for the occurrence of spatial clustering. First, this might be partly due to the livable environments for *C. sinensis* [32]. Sufficient freshwater resources and suitable climate conditions in these regions make sure that different growth states of *C. sinensis* and the competence of infection exist, thus facilitating the spread of clonorchiasis. Second, this fluke develops in two intermediate hosts in fresh water and one definitive host, giving it a clear survival advantage [29, 38]. In these settings, the existence of abundant intermediate hosts and definitive hosts laid good foundations for the formation of a relatively suitable food chain for predation and prey, thus making it possible for *C. sinensis* to develop a complete life history and resulting in clonorchiasis prevalence [3]. Moreover, studies showed that the well-developed fish breeding in these regions and the dietary habit of eating “sashimi” collaboratively promote the transmission of clonorchiasis [32, 39]. In certain areas, poor management of human and animal feces results in their discharge into water sources, potentially containing eggs of *C. sinensis*, which can release miracidia and thus lead to the formation of an endemic disease [3, 29]. Additionally, the high accessibility to infection sources was an important contributor that should not be ignored [3]. It was shown that habits such as catching fish without washing hands, holding fish with the mouth, and mixed use of the kitchen chopping boards could also increase the risk of infection.

This research, consistent with the previous study [17], found that the prevalence rate of clonorchiasis in males was significantly higher than that of females in Guangdong. Males have more chances to eat raw fish and are more indulged in this diet [40]. Although health education has been carried out for a long time, these dietary habits in men were hard to change [3, 17]. Moreover, public officials, medical staff, and teachers had been reported to have higher infection rates among all occupations in this study, which further indicated the paradox between knowledge and behavior where the knowledge of clonorchiasis could not prevent people from consuming raw fish [32]. Additionally, the findings that the infection rate increased with age (0−50 years) and people aged between 40 to 60 years had the largest proportion of *C. sinensis* infection, in line with a previous study [3], indicated that the frequency of raw fish consumption was associated with clonorchiasis. As for education level, people with college degrees reported the highest rate. The confounding effect of age might explain the situation. The direct medical costs for patients with clonorchiasis significantly differed according to the length of stay. Generally, patients with severe clinical symptoms are more likely to have longer stays and higher direct medical costs. As previous findings showed, patients with different complications of *C. sinensis* infection had quite different costs [5].

This study quantified the current disease burden of clonorchiasis in Guangdong by using indexes of prevalence rate, DALYs, and direct medical costs and demonstrated its distribution characteristics and temporal trends. To the best of our knowledge, this was the first study having evaluated the disease burden of clonorchiasis in endemic areas from diverse dimensions and explained the sub-regional and temporal variation.

However, several limitations need to be acknowledged. First, due to the inaccessibility of data on clonorchiasis deaths in Guangdong, this study followed the approach of previous studies [12, 21] and extracted the key parameters from the literature. The parameters used to calculate DALYs were the best available data we could find. Second, in the cost of illness study, because measurement of direct non-medical and indirect costs is not feasible with current data sources, only direct medical costs were included in this analysis. Thus, the economic burden reported in our analysis may be underestimated. It was recommended that the recording and archiving of relevant data should be strengthened in the future to provide more precise information.

## Conclusions

Although effective measures have been taken to prevent and control clonorchiasis, the disease burden caused by clonorchiasis in Guangdong is still remarkable and showed a brief rebound in 2021. This situation highlights the urgent need for sustainable prevention and control strategies. Tailored preventive recommendations should be formulated based on an evidence-based scientific approach, considering the distribution characteristics of the disease burden. In endemic areas, it is crucial to prioritize efforts such as strengthening fish farming management, improving food safet,y and enhancing awareness, which plays a vital role in effectively combating clonorchiasis. It is important to pay particular attention to men, adults, and individuals with lower levels of education during the clonorchiasis prevention campaign.

## Data Availability

The datasets used and/or analyzed during the current study are available from the corresponding author on reasonable request.

## Abbreviations

CNY: Chinese Yuan
YLLs: Adding years of life lost
CPI: Consumer Price Index
WHO: World Health Organization
GBD: Global burden of disease
DALY: Aisability-adjusted life year
CI: Confidence interval
